# Glucagon-like Peptide-1 Receptor Agonists in Treating Diabetes Mellitus and Weight Loss with Potential Application to Rare Obesity-related Genetic Disorders: A Commentary

**DOI:** 10.33696/signaling.7.153

**Published:** 2026

**Authors:** Lucas Francis, Merlin G. Butler

**Affiliations:** 1 Kansas City University College of Osteopathic Medicine, Kansas City, MO 64106, USA; 2 Departments of Psychiatry & Behavioral Sciences and Pediatrics, University of Kansas Medical Center, Kansas City, KS 66160, USA

## Background

Glucagon-like peptide-1 (GLP1) and receptor (GLP1R) agonists are now in common use for treating type 2 diabetes mellitus and weight loss as major global health burdens affecting millions worldwide with prevalence of both diagnosed and undiagnosed diabetes approaching 15% for all U.S. adults increasing with age (www.cdc.gov/diabetes/php/data-research/index.html). More than two in five U.S. adults have obesity (www.cdc.gov/obesity/adult-obesity-facts/index.html) along with a growing number of rare but recognized genetic-related obesity disorders. These include Prader-Willi, Alstrom, Bardet-Biedl, fragile X, Kallmann, Down and Smith-Magenis due to single gene variants, chromosomal anomalies, imprinting, trisomy or triplet repeat expansion defects [1–7]. For example, Prader-Willi syndrome is caused by errors in genomic imprinting, generally from a *de novo* paternal 15q11-q13 deletion and recognized as the most common genetic cause of morbid obesity in childhood [e.g., 3].

Francis and Butler [8] investigated genetic and molecular mechanisms and pathways with web-based *in silico* approaches for GLP1R activation and medication use. They noted effects previously observed along with new observations on metabolism, glucose and insulin production, gastrointestinal motility and related peptides and hormones impacting behavior and satiety related to both type 2 diabetes and obesity. Their approach utilized three separate searchable gene-protein computer-based programs and databases (STRING, Pathway Commons and BioGRID) to analyze functional interactions, pathways and mechanisms by query of GLP1R and related metabolic and appetite regulatory networks associated with disease reportedly involved in the general population for both diabetes and obesity. Their observations may also apply to the treatment of rare obesity-related genetic disorders with their own list of characteristics and unique causes, challenges and barriers [9,10].

Diabetes mellitus is a metabolic disorder characterized by hyperglycemia leading to serious complications contributing to mortality and morbidity such as nephropathy, neurological deficits, retinopathy, coronary heart disease and strokes [11,12]. Type 2 diabetes mellitus accounts for more than 90% of diabetic cases with genetic factors playing important roles [13–19] with weight gain and defective insulin production, secretion and function [17]. Since approval by the FDA in 2017 [12], semaglutide, a glucagon-like peptide-1 (GLP1) receptor (GLP1R) agonist, and other therapeutic agents commonly marketed under brand names Ozempic, Wegovy and Rybelsus are now prescribed to treat type 2 diabetes and weight loss [12,20]. To further study the causes and mechanisms related to type 2 diabetes and identification of pathophysiological findings including genetic-protein factors and their interactive mechanisms for drug selection and treatment, close observation, monitoring and long-term outcome assessments are needed. This will require more time and effort with points addressed in this commentary and discusion of potential treatment for rare obesity-related genetic disorders.

This is an open-access article distributed under the terms of the Creative Commons Attribution License, which permits unrestricted use, distribution, and reproduction in any medium, provided the original author and source are credited.

## Introduction

GLP1R agonists are recognized analogs of endogenous GLP1, a peptide with an incretin effect enhancing insulin secretion after caloric intake [17] and secreted by intestinal L-cells playing an important role in glycemic regulation and satiety signaling in humans [21]. For example, semaglutide mimics physiological effects that lead to weight reduction by acting on appetite control at the brain level including the hypothalamus and brainstem [22,23]. Hence, GLP1R agonists are new therapeutic agents but may have unexpected neurological changes and retinopathy along with gallbladder and cardiovascular issues [23]. A better understanding of the genetic status may advance optimal medical care, treatment success and positive outcomes for affected patients as addressed in this commentary.

Overlapping mechanisms which undoubtedly relate to optimal drug action and dosage for treatment success may depend on the patient’s biology and genetics-related human diversity with response impacted by mechanisms of action, processes and disease state with or without disease progression. Functional mechanisms might be altered or influenced by the dosage prescribed for GLP1 or GLP1R agonists and the patient’s overall health status. In addition, specific gene variants with heterogeneity could lead to biological disturbances and response to medication influencing treatment success. Furthermore, genetic and/or protein testing and monitoring GLP1R agonist response may be considered for those with type 2 diabetes and obesity. However, a positive response to short-term GLP1 or GLP1R agonist treatment does occur but exceptions are noted requiring more studies to identify biological markers or predictors for success.

Currently, agonist binding to GLP1 receptor protein encoded by the *GLP1R* gene leads to inhibition of glucagon secretion, increased insulin release and delayed gastric emptying with decreased appetite. These effects stimulate adenylyl cyclase and G protein signaling for promotion of insulin synthesis and release [18,23–25]. Hence, normal *GLP1* and *GLP1R* genes and their encoded proteins with related biological processes, pathways and functional mechanisms are apparently required to treat patients successfully and may be a focus of potentially new investigations in humans with the continued use of GLP1R agonists. For example, an *in silico* analysis of GLP1R was reported by Francis and Butler [8] using updated integrated web-based programs and databases for identification of existing gene and protein related interactions that affect recognized pathways, molecular functions, biological processes and associated diseases. This web-based integrated genetic and protein mechanistic approach to study GLP1R impact was stimulated by GLP1R agonist drugs often prescribed with a growing need to treat type 2 diabetes and obesity.

## Review of Data Strategy and Methodology

The methodology used and reported results by Francis and Butler [8] became a focus of this commentary. They searched literature (e.g., PUBMED) and other sources along with computer web-based programs and databases by focusing on keywords such as “glucagon-like peptide-1 and receptor (GLP1R)”, “glucagon”, “protein and gene defects/variants” and “obesity.” Other resources included Online Mendelian Inheritance in Man (www.omim.org), Ensembl (www.ensembl.org), Gene Cards (www.genecards.org) and Gene Reviews (www.genereviews.org). For example, the searchable STRING web-based integrated program and database (https://string-db.org) [e.g., 26,27] predicts protein-protein associations along with their functional mechanisms and biological networks to record the top biological processes, molecular functions, cellular components, pathways, and disease-gene associations when searching GLP1 or GLP1R proteins and their role in drug action.

The web-based programs compile, store and integrate curated updated information with documented experimental data to identify and formulate computational predictions of objective comprehensive protein networks. This process encompasses both physical interactions and functions for statistical analysis and generation of figures to illustrate existing related data and display curated results. The interactions could be both direct (physical) or indirect (functional) protein associations and derived from genomic context predictions, high-throughput laboratory experiments, automated text mining from literature sources and other databases including conserved co-expression patterns of genes. The web-based program and statistical approach analyzes targeted genes and their encoded proteins. Identification of potential regulatory protein nodes were characterized with functional clusters and signaling cascades relevant for gene expression and disease mechanisms.

Four separate analytical criteria were used by the STRING web-based program including ***Count in network*** which indicates how many proteins were in the visualized network with annotations utilizing a particular term. ***Strength*** describes the size of the enrichment effect in a visualized network regarding the number of proteins expected to be annotated in a random network of the same size. ***False discovery rate (FDR)*** examines the significance of enrichment to conceptualize the rate of type I (false positive) errors in the null hypothesis with reported p-values corrected for multiple testing. ***Signal*** is defined as a weighted harmonic mean between the observed/expected ratio and −log(FDR) and meant to balance the metrics of larger and smaller terms for ordering enriched terms (see https://string-db.org for more in-depth information and cited literature sources [26,27]). These approaches thereby provide an updated systematic method to evaluate protein-protein interactions, shared biological roles, molecular functions and recorded pathways with disease associations to gain an understanding of disease, processes and pathology required for development of potential therapeutic agents.

Two other searchable web-based programs were Pathway Commons (www.pathwaycommons.org/) for gene-gene regulatory networks and functional interactions and Biological General Repository for Interaction Datasets (BioGRID) (https://thebiogid.org/) for identification and validation of functional protein-protein interactive networks. These gene or protein integrations impacted both binding and co-expression or shared processes with a focus on biological and molecular mechanisms for insight into conserved networks and pathways relevant to health and disease. For example, type 2 diabetes and obesity were specifically examined on how GLP1R agonists or related therapeutic agents could treat specific clinical findings including appetite control and metabolic problems with mechanisms of action.

## Summary of Reviewed Results

The STRING results analyzed consisted of ten recognzed protein-protein interactions termed first-tier assessment and their networks. The proteins identified were gastric inhibitory peptide (GIP) and vasoactive intestinal peptide (VIP), both involved in the gastrointestinal system. In addition, glucagon (GCG), a counterregulatory hormone of insulin and guanine nucleotide-binding with related G-proteins (GNAS, GNB1) were identified as key in hormone sensitivity and diabetes mellitus. Pro-opiomelanocortin (*POMC*) was recorded as a separate but important obesity-related gene that encodes this large complex protein hosting individual cleaved peptides (e.g., melanocyte-stimulating hormone, adrenocorticotropic hormone, beta-endorphin and lipotropin) that control adrenal gland function, pain, pigment production, fat metabolism and regulation of hypothalamic-based eating behavior specifically the satiety center [e.g., 3–7]. Additionally, pituitary adenylate cyclase-activating polypeptide 1 (ADCYAP1) was found as a multifactorial regulator of the nervous system and neuronal protection of stress in response to insulin secretion from pancreatic beta cells. Furthermore, the natriuretic peptide precursor A (NPPA) was identified which acts in natriuresis, diuresis and vasodilation by playing a role in diabetes and clinical presentation along with glucagon-like peptide 2 receptor mediated by G proteins and adenylyl cyclase receptor (GLP2R).

The top STRING predicted GLP1R activity included in biological process was adenylate cyclase-activating G protein-coupled receptor signaling pathway; glucagon receptor activity for molecular function; cAMP signaling for KEGG pathway; glucagon-type ligand receptors for Reactome pathway and hypo- and hyperglycemia for disease-gene associations. Figure 1 was obtained and credited from the STRING analytical protein-protein interactive network including components of biological processes [e.g., 26,27] with enrichment of GLP1R agonist activity impacting both type 2 diabetes treatment and weight loss.

The BioGRID protein interactive program and Gene Ontology biological processes related to GLP1R activity found multiple factors for activation of adenylate cyclase activity; cAMP mediated signal impacting energy reserve and metabolic processes; regulation of cytosolic calcium concentration and insulin secretion with small molecule metabolic and cellular actions. For Gene Ontology molecular functions, transmembrane signaling receptor activity was identified and plasma membrane for the Gene Ontology cellular components.

The Pathway Commons program for gene-gene interactions identified estrogen receptor (ESR1), chorionic gonadotropin beta polypeptide 3, 5 and 8 (CGB3, CGB5, CGB8), histamine H2 receptor (HRH2) required for stimulation of gastric acid secretion, lutropin subunit B (LHB) or luteinizing hormone beta subunit, glucagon (GCG) and cannabinoid receptors 1 and 2 (CNR1 and CNR2) involved in eating behavior along with calcitonin (CALCA) and regulatation of serum calcium. Additional targeted data showed that GNAS plays an important role in hormone regulation, response and sensitivity as a complex imprinted locus influenced by parent of origin producing multiple transcripts with alternative promoters. The most often recognized transcript was Gs-alpha subunit as a stimulatory guanine nucleotide-binding G-protein with essential roles in several physiological processes.

Both GNAS and GCG are encoded proteins involved in systemic hormone regulation, specifically for glucose production and eating behavior as key factors in type 2 diabetes and obesity. Other genes found encoded neuropeptides, transmitters and receptors impacting short and long-term brain function and behavior (e.g., serotonin, dopamine, cannabinoid receptors) and inflammatory markers (e.g., cytokines, interferon) that potentially involve infections. Similarly, insulin resistance in obesity may relate to elevated levels of non-esterified fatty acids, cytokines, hormones and inflammation [28]. These may be key in connecting impaired pancreatic beta cell function, insulin levels and resistance with inflammation seen in type 2 diabetes and comorbidities during disease progression [29]. Thus, several proteins and hormones regulate growth and development, appetite, energy and metabolism involved in obesity.

## Discussion

### Type 2 diabetes and obesity

The combined results from the analytical study by Francis and Butler [8] suggested meaningful roles for GLP1R-associated genes and their encoded proteins with functions in regulation of intestinal motility and vasoactivity, smooth muscle relaxation of the gastrointestinal tract and promotion of vasodilation with slow intestinal transit. These actions align with GLP1R agonists by delaying gastric emptying and mechanisms to prolong satiety and support weight loss. Additionally, the adenylate cyclase-modulating G protein-coupled receptor signaling pathway supports increased intracellular cAMP levels impacting energy and activation. These pathways and molecular functions underscore the gastrointestinal effects of GLP1R agonists and ability to reduce gut motility with altered nutrient absorption influencing weight gain.

Both obesity and type 2 diabetes mellitus share genetic-pathological mechanisms involving hundreds of implicated genes [13,18,19,30] with about 400 obesity-related genes identified from the literature and located on chromosome ideograms by Butler and colleagues [31]. In addition, over 200 genome-wide expression studies in type 2 diabetes using adipocytes from lean, obese and type 2 diabetic subjects found about 2000 differentially expressed unique genes with potential to impact both type 2 diabetic and obese subgroups [18]. The gene enriched terms found related to lipids, cell death and differentiation, regulation of insulin and phosphorylation, fatty acid transport, glutamate receptor binding and response to individual hormone and glucose levels [32]. Earlier studies showed an impact on adipogenesis, signal transduction by G protein-coupled receptors and lipid metabolism with specific involvement of insulin-related genes (e.g., *IGF1, INS, IRS1*), feeding behavior, cholesterol metabolism, glucose and cholesterol homeostasis pathways and receptor binding effects on glucose homeostasis, body weight and circulating insulin and triglyceride levels [33]. *PPARG, UCP3, ENPP1, POMC* and *FTO* obesity-related genes [3–5,31] were consistently identified and showed key molecular-related mechanisms and factors impacting coding (exon) and non-coding (miRNA) expression patterns in syndromic obesity [34]. If these and other recognized obesity and/or diabetic genes are altered, then a disease-state may or may not respond to conventional drug treatment leading to potential side-effects or ineffective treatment. These observations were similarly reported by Francis and Butler [8] using their computational biology approach when studying GLP1R.

Gene response and altered encoded protein could demonstrate inter-patient diversity in disease causation and progression for both type 2 diabetes and obesity affecting treatment. Therefore, genetic testing may help in identifying and addressing diversity to possibly improve selection and treatment approaches for favorable outcomes. Currently, whole exome/genomic DNA sequencing and/or disease specific gene panels are readily avaialable with bioinformatics to identify causative or implicated genes impacting treatment options. Therefore, a proposed genetic testing approach could lead to a more comprehensive individualized care and treatment plan for drug selection and disease surveillance. Ideally, understanding the diverse genetics, biology and pathophysiology of these diseases may recommend treatment strategies to address specific needs, and conversely, development of treatment registries, recording natural history and family pedigree data should be encouraged to better understand the role of genetics in both type 2 diabetes and obesity with treatment respnse.

Drug outcome responses should be further monitored as drug mechanism of action may depend on the biology including genetics and related protein functions directly complicated by genetic diversity or gene variants per patient. Furthermore, existing or new disease comorbidities with or without a positive family history could impact drug selection, response and quality-of-life when treating type 2 diabetes and/or obesity, particularly if unexpected clinical observations are noted that contribute to drug success. Hence, therapeutic agents should be monitored and responses addressed accordingly to identify unforeseen factors that could alter optimal care and continued use. Lastly, pharmacogenetics testing would be encouraged prior to selecting and managing medications, as disease severity, mechanisms and progression could be affected by genetics, the disease status, comorbidities and other medications thereby altering drug response.

### Rare obesity-related genetic disorders

Obesity is a complex disease with multifactorial causation including both genetic and environmental factors. Significant public health problems and comorbidities include type 2 diabetes, hypertension, obstructive sleep apnea, cancers, cardiovascular diseases, and depression. Several factors contribute to obesity including readily available dense food sources, decreased physical activity and sedentary lifestyles, decreased overall energy expenditure and lastly the role of genetics and human diversity [3,5,31,33–35]. Advances in genomic technology will allow identification of gene variants related to diseases with obesity and involvement of the nervous system with regulation of energy utilization, expenditure or storage, important to gain an understanding of the biology. Furthermore, hormone production and gastro-neuroendocrine peptides that control eating behavior may be affected by specific protein transporters or receptors altering overall energy utilization, consumption and homeostasis [36]. These key factors are most likely under genetic control [3,5,31,35,37,38] as heritability estimates indicate that genetic factors contribute up to 70% of obesity [39,40].

Hundreds of recognized obesity genes primarily affect neuropeptides and genetic pathways for weight gain, lipid production, metabolism and transport in utilization and fat deposition for food selection, patterns and eating behavior. Some of these genes include ghrelin and leptin with receptors involved in stimulating or inhibiting food consumption, along with hormones such as insulin and glucagon. Other genes affect muscle cells, their structure, quantity and function which alters physical activity, energy expenditure, storage and utilization. Several of these factors are found in rare obesity-related genetic disorders [e.g., 3,4].

Dozens of syndromic obesity-related genetic disorders are recognized that play an underreported and understudied role in obesity and overall health. It is estimated that over 30 neuroendocrine peptides alone exist that play a role at the brain level or peripheral sgnaling of appetite regulation when disturbed leading to obesity [e.g., 3–5]. Syndromic examples in humans include Prader-Willi, fragile X, Kallmann, Bardet-Biedl, Smith-Magenis, Alstrom, Carpenter, Down and disorders of GNAS inactivation [2–5,41–43].

The causation of syndromic obesity is due to a variety of genetic defects including single gene variants. For example, Alstrom syndrome is due to a disturbed recessive *ALMS1* gene with clinical findings of cone-rod dystrophy, deafness, cardiomyopathy, kidney disease and truncal obesity during the first year of life. Bardet-Biedel syndrome (BBS) consists of several types including recessive genes (e.g., *BBS1, BBS2*, etc.) [2–5,42]. Features in BBS include abnormal retinal pigmentation, polydactyly, cognitive impairment, seizures, hypogonadism, kidney problems and central obesity in the first year of life. Gene copy number disturbances (e.g., chromosome deletions or duplications) are noted in obesity-related disorders including Smith-Magenis syndrome with a 17p11.2 deletion and involvement of the dominant *RAI1* gene leading to altered circadian rhthym and hypothalamic dysfunction. When *RAI1* is deleted in mice then down-regulated *BDNF* (brain derived neurotrophic factor) levels occur contributing to hyperphagia, obesity, altered fat distribution and abnormal behavior [44]. Smith-Magenis syndrome is characterized by distinctive facial features with a broad, flat midface and brachycephaly, brachydactyly, speech delay, hypotonia, developmental delay, sleep dissturbances, self-injurious behavior and childhood onset truncal obesity [2–5].

Other genetic defects include genomic imprinting errors seen in Prader-Willi syndrome (PWS), the most commonly recognized genetic cause of childhood obesity, generally due to a paternal deletion of the 15q11-q13 region involving imprinted genes/transcripts such as *MAGEL2* and *SNRPN1* [3,7,41,43]. PWS is characterized by severe infantile hypotonia, poor suck and feeding difficulties, hypogonadism, short stature, small hands and feet due to growth and other hormone deficiences, developmental delay, characteristic behavior problems (e.g., skin picking) and childhood onset hyperphagia with subsequent obesity, if not externally controlled. Triplet CGG repeat expansion defects occur in the X-linked *FMR1* gene in fragile X syndrome that encodes a selective RNA-binding protein complex associated with polyribosomes, suggesting involvement in protein translation for brain development and function [4]. Their clincal features include intellectual disability, autism, behavioral concerns, mild connective tissue dysplasia with large appearing ears, macro-orchidism, seizures and sleep disturbances [1–4].

The last category of gene defects is trisomy found in Down syndrome with 16 loci as candidates in energy and rective oxygen species metabolism including *SOD1* (superoxide dismutase 1) and nine loci for brain development, neuronal loss and neuropathology such as the *APP* gene encoding amyloid beta (A4) precursor and *RCAN1* as a regulator of calcineurin with six loci involved in folate and methyl group metabolism [e.g., cystathionine beta-synthase (*CBS*)]. Trisomy 21 is characterized by hypotonia, intellectual disability, short stature, flat facies, slanted palpebral fissues, small ears, congenital heart defects, endocrine dysfunction and obesity [1–4,31,35,37].

More funded research is needed in obesity, particularly in genetics and the worldwide epidemic. Investigations of at-risk genetic factors and syndromic obesity should lead to a better understanding of molecular genetic mechanisms and their protein interactions applicable for simple or non-syndromic obesity in the general population. Advanced genetic laboratory testing now includes whole genomic sequencing to identify large or small genetic defects and gene variant types supported by proteomics to better characterize disturbed molecular mechanisms and disease relationships of specific gene(s) leading to potential genomic-driven medical care. This process would be applicable to both treating syndromic or non-syndromic causes of obesity and the genetic basis of those affected with rare obesity-related genetic disorders and elucidate specific genetic and biological underpinnings for discovery and treatment. Furthermore, genotype-phenotype correlations with use of computational biology may allow identifications of disturbed biological processes, pathways, inheritance patterns and molecular mechanisms for gain or loss of gene function. This information may play a role in controlling energy expenditure, physical activity, muscle mass and strength, hormones, metabolism, eating behavior and regulation of appetite with balance of energy utilization thereby having a direct application in the general population when studying rare obesity-related genetic disorders.

Of interest is a report by Correia *et al.* [45] in Smith-Magenis syndrome (SMS) with involvement of the *RAI1* gene encoding retinoic acid-induced 1 protein implicated in glucose and lipid metabolism utilizing the MC4R pathways and relationship with POMC important in regulating eating behavior in humans. They reported their experience in weight management of a challenging patient with SMS and use of GLP-1 receptor agonists. The female patient experienced significant weight gain from early childhood and developed complications from type 2 diabetes, dyslipidemia and steatotic liver disease. Initial weight management efforts were unsuccessful and at 18 years, subcutaneous semaglutide was introduced. This resulted in marked improvement in impulsivity, food cravings and weight control. Their report underscores GLP1R agonists in managing both obesity and behavioral symptoms in Smith-Magenis syndrome.

GLP1R agonists were also administered in clinical trials in Prader-Willi syndrome [10,46–48]. For example, a report by Ng *et al*. [48] summarizing ten published studies and use of GLP1R agonists in 23 subjects with PWS and 70 percent had type 2 diabetes. They found improvement in body mass index in 10 of the 23 patients, reduction in HbA1c levels in 19 cases and all 23 subjects reported appetite or satiety improvement. There were no reported serious side effects in this genetic obesity-related disorder and biological processes impacted or altered by errors of genomic impriting including *MAGEL2* and *SNRPN* genes/transcripts [7]. These genes/transcripts affect mechanisms of action including RNA splicing and regulation, circadian rhythm, ubiquitin ligase activity and protein degradation including hormones and apoptosis. Small GTP-binding proteins were found that impact cellular events and growth, cytoskeletal reorganization and protein kinase activation for neurodevelopment and immunodeficiency [e.g., 7,10]. Further studies are needed to identify the effectiveness of these agonists and dosage levels for both short- and long-term management in those with genetic syndromes and obesity.

## Conclusions

### Type 2 diabetes and obesity

The report by Francis and Butler [8] utilized three separate web-based programs and databases to analyze the *GLP1R* gene and its encoded protein-protein interaction, molecular mechanisms, biological processes and associated diseases. They identified and summarized existing information, genetic factors and mechanisms of action involved in GLP1R agonists for treating type 2 diabetes and obesity. The GLP1R encoded protein networks orchestrate complex signaling mechanisms, hormones, substrates and pathways affecting integrated gene and protein interactions. These agonists do appear to improve glycemic control, important in type 2 diabetes and promote weight loss with normalization of glucose levels via metabolic, endocrine, gastrointestinal and functional interactive pathways. However, more research is needed to monitor both short- and long-term outcomes, side effects and comorbidities.

Adenylate cyclase-modulating G protein-coupled receptor and signaling pathways were consistently identified across the first-tier to third-tier STRING level including up to 30 genes and their encoded proteins which are further supported by two separate interactive gene and/or protein web-based programs for validation and characterization of GLP1R agonist activity. Other studied genes and/or proteins found of importance included GIP, GNAS, GCG, SCT, POMC, VIP, GLP1 and ADCYAP1R, some identified for the first time (e.g., GNAS, CALCA, CALCB, HRH2, CNR1, CNR2) that play a role in hormone control and sensitivity, electrolyte balance and neurological/behavioral findings, specifically, GNAS and GNAS-related disorders with hormone insensitivity and obesity [e.g. 2,43]. In addition, alterations in polypeptide POMC cleavage and function in Prader-Willi syndrome (PWS) showed deficiencies in prohormone convertase PC1 (encoded by *PCSK1*) and impaired POMC prohormone processing of individual peptide production involved in eating behavioir [49]. Both humans and mice deficient in PC1 display hyperphagic obesity, hypogoandism, decreased growth hormone and hypoinsulinemic diabetes from impaired prohormone processing with individual hormones and peptides cleaved from the large complex POMC protein suggesting major neuroendocrine features in PWS may result from PC1 deficiency [49]. The combination of overall actions identified in the *in silico* study reported by Francis and Butler [8] include glucose regulation, endocrine function, insulin production and secretion, hypothalamic-based satiety control, gastrointestinal motility, electrolytes and other related peptides that control metabolism, energy expenditure and eating behavior with related neuropeptides and hormones.

Other organs and tissues could be further affected including skeleton, muscle mass and strength and endocrine glands for sex, thyroid and adrenal function with involvement of neuropeptides and related brain regions. Conversely, GLP1R signaling cascades described the activation and increased intracellular cyclic AMP that collectively alters glucagon production, insulin secretion, glycogenolysis, vasoactive intestinal peptide and other gut-endocrine related peptides and hormones needed for satiety control.

Additional factors found were obesity-related peptides that impact hormone function and signaling (e.g., POMC), renin, electrolytes and diuresis, insulin secretion and circadian rhythm with other behaviors. These associations and interactions range from hypoglycemia to broader endocrine dysfunction, specifically identifying a relationship with GNAS affecting growth and response, electrolytes, skeletal disturbances and related hormone sensitivity patterns. Understanding these established and potentially new interactions with the role of genetics and human diversity in medication response should be further investigated. Potential long-term systemic effects should be monitored in treatment protocols adjusted accordingly, along-with medication resistance or loss of efficacy. The study of potential gene variants for susceptibility of type 2 diabetes and/obesity and their status could provide further insight in treatment, selection, response and outcome.

In summary, the recognition of molecular functions and protein insights should enhance our understanding of therapeutic options, their mechanisms and related clinical expectations. Genetic-related findings when altered could impact clinical outcomes and treatment driven in humans by genetic heterogeneity. Potential or unforeseen systemic effects with long-term use of new drugs and/or resistance risks are unknown. Long-term effects of teatment on organ systems are unknown or their impact on calcium and electrolytes, gut peptide and hormone levels, vision or changes related to muscle and bone mass or strength.

As with most studies using *in silico* analyses, limitations are noted when searching existing data from literature or other sources as new information is reported or learned daily. Futhermore, more studies are needed with new analyses to further impact GLP1R or related agonist use, both short- and long- term effects and related functions, their molecular mechanisms and therapy protocols. The use of related treatment registries is encouraged for recording natural history data, genetic testing results and other factors that may impact short and long-term drug response, outcomes and measures including resistance or unexpected comorbidities with other medications and unexpected clinical patterns. The enriched functional molecular mechanisms and protein interactions described and questions raised in this commentary may impact disease and response to treatment. The usage of new therapeutic agents and medical care findings, should be continuously monitored as standard for success and to identify unforeseen factors addressed for optimal care.

### Rare obesity-related genetic disorders and use of GLP1 and GLP1 receptor (GLP1R) agonist treatment, barriers and limitations

The growing list of barriers and limitations with experiences of clinical trials in rare genetic diseases with obesity will require further investigations as rare diseases are defined as affecting fewer than 200,000 individuals per disease in the United States and no more than one in 2000 persons by the World Health Organization [e.g., 9,10]. Providing medical care and developing novel treatment options for patients with a rare disease are challenging, including those disorders having a variety of genetic defects. The rarity of a condition further complicates awareness and knowledge with extra efforts needed to obtain an adequate number of subjects for clinical trials, enrollment and confirmation of syndromic specific genetic defects in relationship to genetic heterogeneity. Understanding the biology and clinical findings related to specific gene defects in rare disorders would be crucial in development of therapeutic agents for use in clinical trials. Therefore, the recruitment of participants worldwide with language barriers and customs and access to medical care, needed resources and available testing options are challenging. Different age groups and complicating factors related to rare disorders make it difficult to recruit and enroll in clinical trials including lack of natural history data. Although these obstacles are present, the study of rare obesity-related genetic disorders should be pursued and potential use of new medications such as GLP1 or GLP1R agonists with larger number of subjects. The early results in treating obesity in rare genetic obesity-related diseases with GLP1 related agonists as described are encouraging with genes/proteins found in common playing a role in non-syndromic obesity specifically for POMC, GNAS, GCG and potentially others. These results may have a direct application in understanding and treating individuals with type 2 diabetes and obesity in the general population.

## Figures and Tables

**Figure 1.**
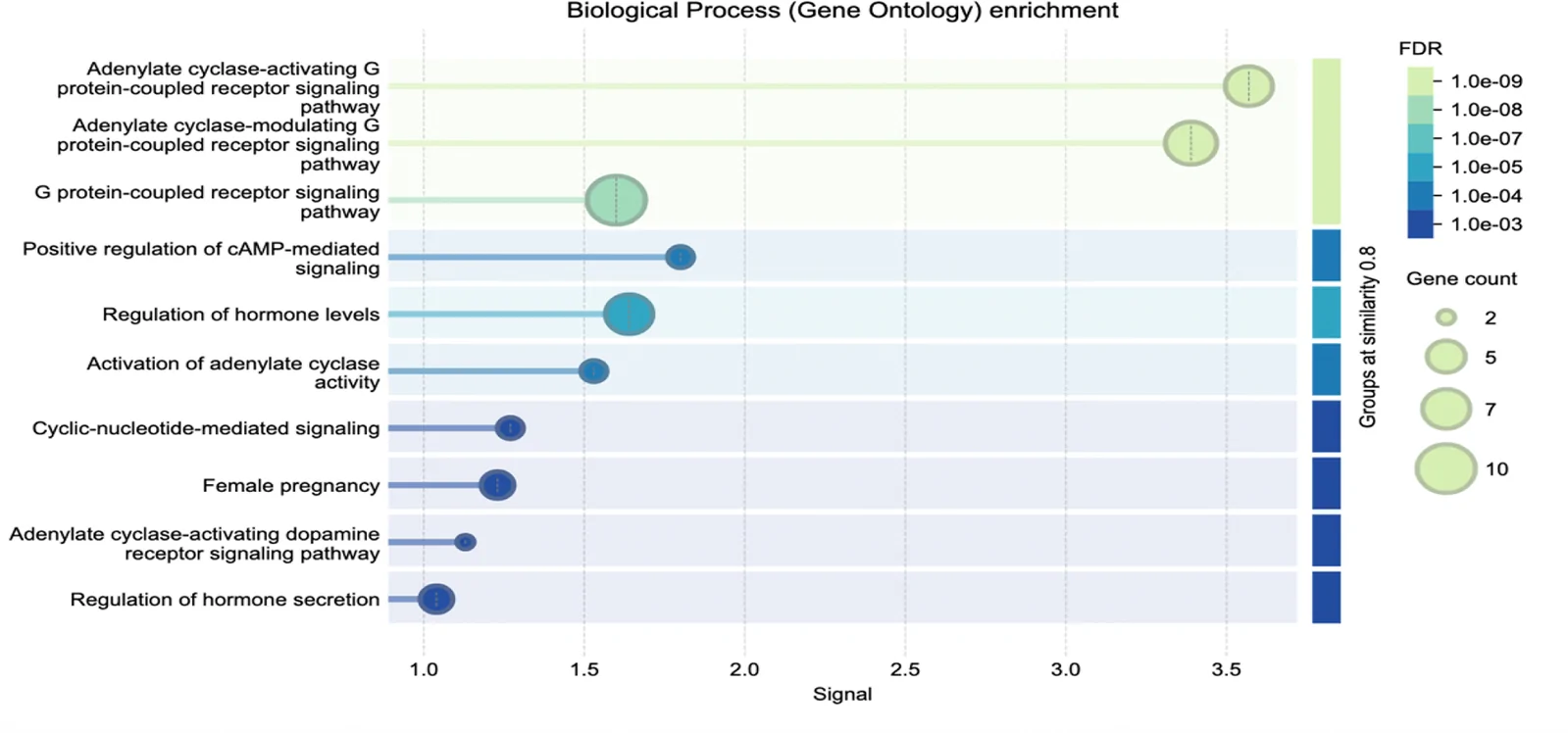
Biological processes (Gene Ontology) from the literature and identified with the searchable STRING web-based program and analysis (www.string-db.org) using ten inter-related proteins termed first-tier for GLP1R functional protein, signal intensity, gene count and false discovery rate [e.g., 26,27].

## Data Availability

The original contributions addressed and presented in this commentary are included in the article.
